# Corrigendum to ‘SPiQE: An automated analytical tool for detecting and characterising fasciculations in amyotrophic lateral sclerosis’ [Clin. Neurophysiol. 130 (2019) 1083–1090]

**DOI:** 10.1016/j.clinph.2019.10.004

**Published:** 2020-01

**Authors:** J. Bashford, A. Wickham, R. Iniesta, E. Drakakis, M. Boutelle, K. Mills, C. Shaw

**Affiliations:** a UK Dementia Research Institute, Department of Basic and Clinical Neuroscience, Maurice Wohl Clinical Neuroscience Institute, Institute of Psychiatry, Psychology and Neuroscience, King’s College London, United Kingdom; b Department of Bioengineering, Imperial College London, United Kingdom; c Department of Biostatistics and Health Informatics, King’s College London, United Kingdom

The authors regret the following errors, which appear in their published article:

1. On page 2 (p1084), section 2.4, 1st para, line 3, the text currently reads: “A bandpass filter (20–500 Hz) was applied without notch filtering.” It should read: “A bandpass filter (20–500 Hz) was applied with 50 Hz notch filtering.”
2. On page 6 (p1088), section 4 (Discussion), paras 4 (line 8) and 5 (line 1): the published references to *Fig. 4* are wrong and in both instances should make reference to *Fig. 3*.

The authors would like to apologise for any inconvenience caused.

